# PW03-019 – Survey of off-label ANTI-IL1 treatments in France

**DOI:** 10.1186/1546-0096-11-S1-A245

**Published:** 2013-11-08

**Authors:** L Rossi-Semerano, B Fautrel, D Wendling, E Hachulla, A Meyer, S Ottaviani, C Galeotti, M Fouillet-Desjonqueres, O Richer, I Touitou, I Koné-Paut

**Affiliations:** 1 Department of Paediatrics and Paediatric Rheumatology, Bicêtre Hospital, National Reference Centre for Auto-inflammatory Diseases, Le Kremlin Bicêtre, France; 2 Department of Rheumatology, Hôpital La Pitié Salpêtrière, Paris, France; 3 Departement of Rheumatology, CHU Besançon, Besançon, France; 4 Service de Médecine Interne, Centre de Référence des Maladies Auto-immunes et Maladies Systémiques rares, CHRU Lille, Lille, France; 5 Service de Rhumatologie, Hôpitaux universitaires de Strasbourg, Hôpital de Hautepierre, Strasbourg, France; 6 Service de Rhumatologie, CHU Bichat, Paris, France; 7 Service de Pédiatrie Générale et Néphrologie Pédiatrique, Hôpital Femme Mère Enfant, CHU Lyon, Lyon, France; 8 Service de Pédiatrie Médicale, CHU Bordeaux, Bordeaux, France; 9 Maladies Auto-Inflammatoires, Laboratoire de Génétique, Hôpital A de Villeneuve, CHRU Montpellier, Montpellier, France

## Introduction

Despite their limited licensed indications, anti-IL1 agents are often used in real-life practice for an increasing number of diseases. A national survey to record their off-label use in France was started in January 2011. The survey is coordinated by the French National Reference Centre for Auto-inflammatory Diseases, under the aegis of the “Club Rhumatisme et Inflammation” (CRI).

## Objectives

The survey aims to gather information concerning: the number of patients treated with anti-IL1 agents in France, the treated disease, the kind and the indication of the used anti-IL1 agents, their efficacy and safety.

## Methods

We set up a physician-directed questionnaire available on the website of CRI since January 2011, covering the following areas: patient data, disease data, anti-IL1 agent, its efficacy, adverse events. We advertised the study on the occasion of French and European rheumatology congresses and by e-mail to French physicians that could be interested. Any adult or paediatric patient who had received an anti-IL1 agent after January 2005 in France could be included after medical informed consent.

## Results

At two years 188 patients (99males, 88 females, mean age 35.2 years), from 37 centres have been included. Main diseases were: adult onset Still’s disease (AoSD) (35), systemic onset juvenile idiopathic arthritis (SoJIA) (29), gout (26), anakinra-treated cryopirin associated periodic syndrome (CAPS) (21), mevalonate kinase deficiency (MKD) (14), familial Mediterranean fever (FMF) (12), SAPHO syndrome (9), Schnitzler’s syndrome (7).

The main off-label used agent was anakinra, used at least once in 182 patients. Canakinumab was used in 23 patients. Rilonacept is not yet available in France. Anakinra shows partial to complete efficacy in most patients (90%); complete clinical response rates vary according to specific diseases, being higher in Schnitlzler’s syndrome, gout, CAPS, AoSD and SoJIA. Fifty four percent of patients showed at least one adverse event (AE), mainly minor injection site reactions, and some showed a serious AE (SAE), mainly severe infection. Preliminary data of our survey suggest that canakinumab was generally well tolerated, without any SAE.

## Conclusion

Two-year results of the survey confirm the wide use of anti-IL1 agents in clinical practice. The main off-label used agent was anakinra**,** which showed efficacy in the vast majority of patients. Patients with Schnitlzler’s syndrome, gout, CAPS and AoSD showed the higher complete clinical response rate. A sizeable number of adverse events, namely injection site reactions, was reported in patients treated by anakinra, Canakinumab was generally well tolerated, without any SAE. The number of patients treated by canakinumab was too small to evaluate its efficacy.

## Disclosure of interest

L. Rossi-Semerano: None declared, B. Fautrel: None declared, D. Wendling: None declared, E. Hachulla Consultant for: Consultant fee from Novartis, Sweedish Orphan Biovitrum, A. Meyer: None declared, S. Ottaviani: None declared, C. Galeotti: None declared, M. Fouillet-Desjonqueres: None declared, O. Richer: None declared, I. Touitou: None declared, I. Koné-Paut Grant / Research Support from: Educational and research grant from Sweedish Orphan Biovitrum, Consultant for: Consultant fee from Novartis

